# Stability of mRNA/DNA and DNA/DNA Duplexes Affects mRNA Transcription

**DOI:** 10.1371/journal.pone.0000290

**Published:** 2007-03-14

**Authors:** Rayna I. Kraeva, Dragomir B. Krastev, Assen Roguev, Anna Ivanova, Marina N. Nedelcheva-Veleva, Stoyno S. Stoynov

**Affiliations:** 1 Institute of Molecular Biology, Bulgarian Academy of Sciences, Sofia, Bulgaria; Lehigh University, United States of America

## Abstract

Nucleic acids, due to their structural and chemical properties, can form double-stranded secondary structures that assist the transfer of genetic information and can modulate gene expression. However, the nucleotide sequence alone is insufficient in explaining phenomena like intron-exon recognition during RNA processing. This raises the question whether nucleic acids are endowed with other attributes that can contribute to their biological functions. In this work, we present a calculation of thermodynamic stability of DNA/DNA and mRNA/DNA duplexes across the genomes of four species in the genus *Saccharomyces* by nearest-neighbor method. The results show that coding regions are more thermodynamically stable than introns, 3′-untranslated regions and intergenic sequences. Furthermore, open reading frames have more stable sense mRNA/DNA duplexes than the potential antisense duplexes, a property that can aid gene discovery. The lower stability of the DNA/DNA and mRNA/DNA duplexes of 3′-untranslated regions and the higher stability of genes correlates with increased mRNA level. These results suggest that the thermodynamic stability of DNA/DNA and mRNA/DNA duplexes affects mRNA transcription.

## Introduction

In living systems DNA provides information for the synthesis of RNAs and proteins. The secondary structure of nucleic acids through its defined physico-chemical characteristics such as the thermodynamic stability of the pairing between the two strands can influence its biological function. The thermodynamic stability of a polynucleotide duplex is defined as the free energy (ΔG) required to unwind it and can be calculated from the entropy (ΔS) and the enthalpy (ΔH) of the pairing between the adjacent bases using a nearest-neighbor method [1]. Published calorimetric measurement of ΔS and ΔH of all possible nearest-neighbor interactions of DNA/DNA [2] and RNA/DNA [3] duplexes allows for calculation of thermodynamic stability of polynucleotide duplexes with a defined sequence [4]–[6]. In order to elucidate the influence of thermodynamic stability of DNA/DNA and RNA/DNA duplexes on transcription, a genome-wide analysis of thermodynamic stability is required.

In this work we present a genome-wide calculation of DNA thermodynamic stability for four genomes in the genus Saccharomyces, using Kowalski's sliding-window approach [6]. We show that DNA/DNA as well as DNA/RNA duplex stability differs between coding and non-coding regions. The lower stability of the DNA/DNA and mRNA/DNA duplexes of 3′-untranslated regions and the higher stability of genes correlates with increased mRNA level. Moreover, mRNA/DNA duplexes appear to be more stable than the corresponding anti-sense duplexes, allowing prediction of open reading frames. Based on these observations the role of thermodynamic stability on transcription is discussed.

## Results

**Figure 1:**
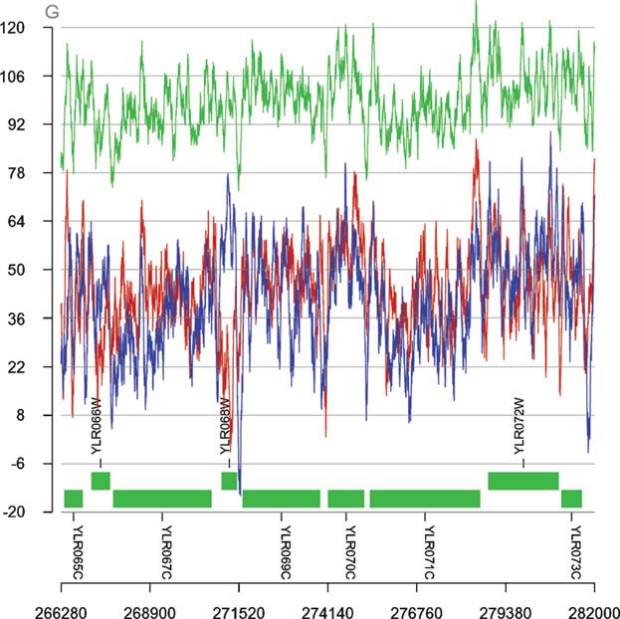
Thermodynamic stability of DNA/DNA (green), sense and antisense RNA/DNA duplexes in a region of chromosome 12 in *S. cerevisiae* (plots of all sixteen chromosomes are available at http://obzor.bio21.bas.bg/stoyno/). ΔG of RNA/DNA duplexes (blue), containing RNA identical to Watson coding strand represents the sense strand for Watson's ORFs and antisense strand for Crick's ORFs. ΔG of RNA/DNA duplexes (red), containing RNA identical to Crick coding strand represents the sense strand for Crick's ORFs and antisense strand for Watson's ORFs.

We created Perl-based software that allowed us to calculate thermodynamic stability of DNA/DNA and RNA/DNA duplexes with arbitrary length using a sliding-window approach. This tool allowed us to calculate the thermodynamic profile over the entire genome of *Saccharomyces cerevisiae* with a step size of 1 bp, and a varying window size (100 bp unless explicitly indicated). Using this set of parameters, the calculated windows' mean value of ΔG of DNA/DNA duplexes for the entire genome is 98.47 kcal/mol. We found that intergenic regions (IGRs) have lower mean values of ΔG average and ΔG minimum (ΔG avg = 92.84 kcal/mol and ΔG min = 78.60 kcal/mol) than genes (ΔG avg = 100.80 kcal/mol and ΔG min = 84.81 kcal/mol) (Figure 1 and Tables S1, S2 and S3).

### DNA/DNA and RNA/DNA duplexes are less stable in 3′-IGRs than in genes

**Figure 2:**
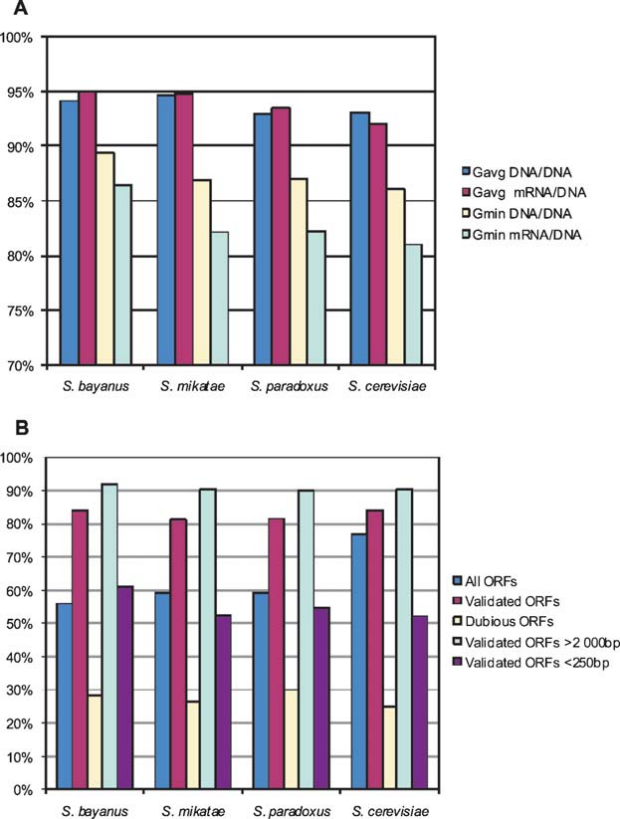
(A) Percentage of ORFs with ΔG values of DNA/DNA and sense mRNA/DNA duplexes higher than ΔG avg and ΔG min of the corresponding 3′-IGRs. (B) Percentage of ORFs with more stable sense than antisense RNA/DNA duplexes as annotated in SGD.

In order to distinguish the roles of the observed differences in duplex stability in transcription initiation and transcription termination, we grouped the intergenic regions into three groups based on the direction of transcription of their neighboring open reading frames (ORFs): (i) IGRs between ORF starts (divergent transcripts), (ii) IGRs between ORF ends (convergent transcripts) and (iii) IGRs between two ORFs transcribed in the same direction (tandem running transcripts) (Table S2). Our results show that IGRs flanked by convergent transcripts have a lower mean value of ΔG min compared to those flanked by divergent transcripts (Tables 1 and S3). These findings are in agreement with previous studies, showing that 3′-termini of several transcription units contain regions prone to unwinding under superhelical stress conditions [7]. To check whether all intergenic sequences are less stable than their adjacent 5′-ORFs, we compared the calculated ΔG values for these two classes of sequences. The results show that out of 6004 ORF/3′-IGR pairs in the *S. cerevisiae* genome 93% of ORFs have a higher ΔG avg and 86% have a higher ΔG min than their corresponding 3′-IGRs (Figure 2A and Tables 2 and S4). To further explore this, we calculated mRNA/DNA duplex stability in the genome of *S. cerevisiae* (see Materials and Methods). As expected, mRNA/DNA duplexes are less stable (that is with lower ΔG) than DNA/DNA duplexes for both ORFs and 3′-IGRs (Table 2). Similar to DNA/DNA duplexes, mRNA/DNA duplexes of 3′-IGRs have a statistically significant lower mean value of ΔG avg than the corresponding genes (Table S3). 92% of the ORFs have a higher ΔG avg and 81% have a higher ΔG min than the IGR adjacent to their 3′-ends (Table S4). Using the available information on the position of the 3′-end processing sites in *S. cerevisiae* [8], we investigated the thermodynamic stability of mRNA/DNA duplexes of the 3′-untraslated regions (3′-UTRs) (window size = 9 bp; see Materials and Methods). 3′-UTRs have statistically significant lower mean value of ΔG than genes (Tables 3, S1 and S3).

**Table 1:** Mean values and standard deviation (in brackets) of ΔG min and ΔG avg of intergenic regions flanked by convergent (→ ←), divergent (← →) and tandem (→ →) running transcripts.

	ΔG min	ΔG avg
	(kcal/mol)	(kcal/mol)
**All IGRs**	78.60 (6.88)	92.84 (6.35)
→ ←	75.66 (6.40)	87.81 (6.19)
← →	81.91 (6.25)	96.33 (4.8)
→ →	78.48 (6.69)	93.73 (5.40)

**Table 2:** Mean values and standard deviation (in brackets) of ΔG min and ΔG avg of genes and their 3′-intergenic regions.

		Genes		Intergenic regions	
		DNA/DNA	mRNA/DNA	DNA/DNA	RNA/DNA
**Mean value ΔG avg (kcal/mol)**	***S.cerevisiae***	100.7 (4.89)	48.78 (7.81)	90.63 (6.53)	32.06 (10.42)
	***S.bayanus***	103.78 (7.31)	53.36 (9.95)	91.71 (7.74)	33.60 (11.76)
	***S.mikatae***	100.24 (4.46)	49.09 (7.26)	89.72 (6.11)	31.93 (9.66)
	***S.paradoxus***	101.18 (4.97)	49.87 (7.40)	90.97 (6.38)	32.67 (10.09)
**Mean value ΔG min (kcal/mol)**	***S.cerevisiae***	84.81 (5.76)	22.97 (11.32)	77.00 (6.68)	8.87 (14.68)
	***S.bayanus***	86.77 (7.00)	27.36 (12.25)	77.13 (7.31)	9.35 (15.36)
	***S.mikatae***	84.46 (5.43)	24.11 (10.30)	76.70 (6.39)	9.92 (13.52)
	***S.paradoxus***	85.19 (5.54)	24.37 (10.57)	77.12 (6.38)	9.41 (13.97)

**Table 3:** Mean values and standard deviation of ΔG avg of sense and antisense RNA/DNA duplexes of genes, introns, exons and 3′-UTRs (window size 9 bp.)

	Mean value of sense ΔG avg (kcal/mol)	Mean value of antisense ΔG avg (kcal/mol)
**All genes**	3.94 (0.68)	3.52 (0.73)
**Genes with introns**	3.71 (0.54)	3.34 (0.57)
**Introns**	2.56 (1.00)	2.87 (0.77)
**Exons**	4.13 (0.59)	3.61 (0.68)
**Genes with annotated 3′-UTRs**	4.07 (0.56)	3.47 (0.64)
**3′-UTRs**	2.70 (1.30)	2.36 (1.49)

### DNA/DNA and RNA/DNA duplexes are more unstable in introns and 3′-end processing regions than in coding sequences

3′–end processing requires several quite degenerate regulatory sequences positioned in the range of 80 nt upstream and 20 nt downstream from the 3′-end processing site [9]–[12]. Therefore, we examined the thermodynamic stability of mRNA/DNA duplexes of these 100 bp 3′-end processing regions (3′-EPRs). Our results showed that the mean value of ΔG of the 3′-regulatory sequences (32.41 kcal/mol) is comparable to the mean value of ΔG avg of the 3′-IGRs and is significantly lower than ΔG avg of the genes (Tables 2 and S3). *S. cerevisiae* genome contains 264 genes with introns. Calculation of introns' thermodynamic profiles (window size of 9 bp) showed that their mRNA/DNA duplexes are significantly less stable than exon's (coding sequences in ORFs) sense mRNA/DNA duplexes (Tables 3 and S1). These results suggest that stable sense duplexes are characteristic of the coding sequences.

### Evolutionary conservation of the thermodynamic pattern

To check if the observed pattern of thermodynamic stability is evolutionarily conserved we calculated the ΔG values of DNA/DNA and mRNA/DNA duplexes for three other related species of the genus *Saccharomyces*-*S. bayanus, S. paradoxus, S. mikatae*, using the available draft genome sequences (Tables S1 and S4) [13]. The averages of ΔG of DNA/DNA and mRNA/DNA duplexes in genes are greater than those in the adjacent 3′-IGR in more than 92% and 93% of the cases, respectively (Figure 2A and Table 2). The minimums of ΔG of DNA/DNA and mRNA/DNA duplexes in genes are greater than those in the adjacent 3′-IGR in more than 86% and 82% of the cases, respectively (Figure 2A and Table S4).

### Correlation between thermodynamic stability of DNA/DNA and RNA/DNA duplexes and mRNA level

We also inspected the possible relationship between mRNA expression level [14] and values of ΔG in genes and their corresponding 100 bp 3′-EPRs. There appears to be a general trend of increased mRNA level with increasing ΔG avg of the ORFs. Spearman's rank correlation coefficients (SCC), assessing the strength of the association between gene's thermodynamic stability and mRNA levels, are 0.209 for DNA/DNA duplexes and 0.142 for mRNA/DNA duplexes (Table S5). Although these values are not particularly high, they bear a strong statistical significance (Table S5). The observed correlations are impressive given that several other factors (like promoter effectiveness, promoter regulation and mRNA half-life) directly influence mRNA level as well. Correlation between stability of coding sequences only and mRNA level is higher: SCC is 0.263 for DNA/DNA duplexes and 0.199 for mRNA/DNA duplexes.

**Figure 3:**
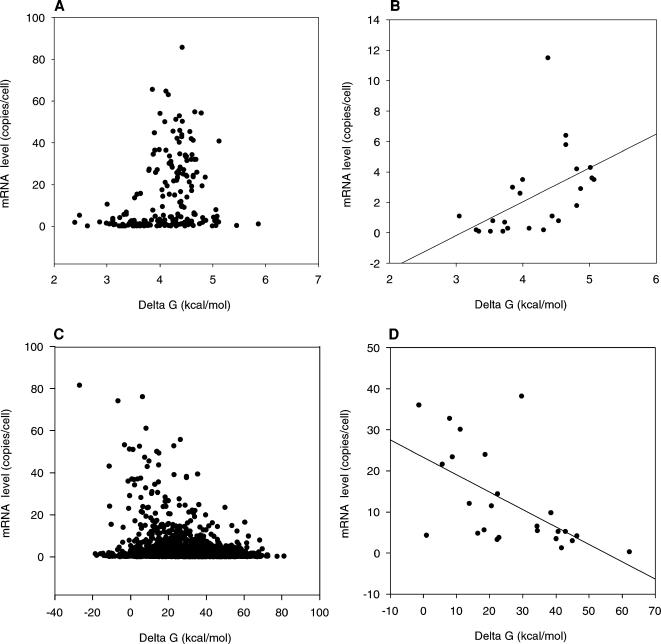
Scatter plot, showing the relationship of mRNA level (copies per cell) and Δ G (kcal/mol) of EPR mRNA/DNA duplexes and Δ G avg of exon mRNA/DNA duplexes. (A) Relationship between mRNA level and Δ G avg of all coding sequences in intron containing ORFs. (B) Relationship between mRNA level and Δ G avg of coding sequences in intron containing ORFs longer than 2000 bp. (C) Relationship between mRNA level and Δ G for all available EPRs. (D) Relationship between mRNA level and ΔG of EPRs for genes shorter than 250 bp.

We next surveyed the relationship between mRNA level and stability of intron-containing genes. In this case we did not find a statistically significant correlation. However, a strong correlation between mRNA level and the stability of the exons was observed: SCC is 0.374 for DNA/DNA duplexes and 0.329 for mRNA/DNA duplexes (Figure 3A and Table S5). The correlation between mRNA level and exon thermodynamic stability increases with increasing ORF length: SCC for intron containing ORFs longer than 2000 bp is 0.658 for DNA/DNA duplexes and 0.691 for mRNA/DNA duplexes (Figure 3B and Table S5). Interestingly, a positive correlation exists between the thermodynamic stability of introns and mRNA level. This correlation increases with increasing ORF length: SCC is 0.611 for DNA/DNA duplexes and 0.560 for mRNA/DNA duplexes. In addition, an inverse relationship exists between mRNA levels and stability of 3′-EPRs. mRNA levels of the ORFs 5′ of the EPR increase with decreasing of 3′-EPR ΔG (Figure 3C) (SCCs are -0.266 for DNA/DNA duplexes and -0.232 for mRNA/DNA duplexes) and this negative correlation rapidly increases with decreasing ORF length. For ORFs shorter than 250 bp SCC is -0.639 for mRNA/DNA duplexes (Table S5 and Figure 3D), indicating strong negative relationship between thermodynamic stability of the 3′-EPR and mRNA level. Similar negative correlation is observed between 3′-UTR's stability and mRNA level (Table S5). The correlations between mRNA level and either ORF's or 3′-EPR's stability suggest a role for the thermodynamic stability in mRNA transcription.

### More stable sense than antisense RNA/DNA duplexes are a common characteristic of the coding sequences

Upon careful scrutiny, the thermodynamic profiles of mRNA/DNA duplexes within genes exhibits yet another interesting feature. There is a strong statistically significant difference between ΔG avg of sense and potential antisense RNA/DNA duplexes in ORFs (Tables 4, S1 and S3). 76.90% of all ORFs have more stable sense mRNA/DNA duplexes than potential antisense RNA/DNA duplexes (Figure 2B). However, the thermodynamic stability of antisense RNA/DNA duplexes positively correlates with mRNA level. Unlike ORFs, the ratio of ΔG avg of potential sense and antisense RNA/DNA duplexes in 3′-IGRs is nearly equal (50.57% of the sense duplexes are more stable than the potential antisense duplexes) (Tables 4 and S4).

**Table 4:** Mean values and standard deviation (in brackets) of ΔG avg of sense and antisense RNA/DNA duplexes of genes and 3′-IGRs and their dependence on ORF length.

		Mean value of sense ΔG avg (kcal/mol)	Mean value of antisense ΔG avg (kcal/mol)
***S. cerevisiae***	**All genes**	48.61 (8.14)	43.41 (8.81)
	**Validated genes**	49.46 (7.17)	42.52 (8.19)
	**Validated genes>2000** bp	47.27 (5.17)	39.97 (6.25)
	**Validated genes<250** bp	44.39 (12.94)	45.81 (11.73)
	**Dubious genes**	42.60 (11.42)	49.75 (10.34)
	**3′-IGRs**	32.06 (10.41)	31.71 (10.31)
***S. bayanus***	**All genes**	51.88 (12.61)	51.45 (13.00)
	**True ORFs**	53.30 (9.90)	46.51 (11.03)
	**Spurious ORFs**	50.50 (14.16)	56.29 (12.96)
***S .mikatae***	**All genes**	46.60 (9.98)	45.60 (9.93)
	**True ORFs**	48.96 (7.49)	42.65 (8.74)
	**Spurious ORFs**	43.21 (11.96)	49.85 (10.01)
***S. paradoxus***	**All genes**	47.41 (9.93)	46.45 (9.82)
	**True ORFs**	49.74 (7.68)	43.57 (8.66)
	**Spurious ORFs**	44.12 (11.67)	50.51 (9.92)

ORFs in the Saccharomyces Genome Database fall into one of the following three categories-verified (experimentally confirmed); uncharacterized (which have orthologs in other species, but without experimental evidence in yeasts to support this); and dubious (without any experimental evidence for their existence). Although dubious ORFs are unlikely to encode a protein, there are no characteristic features to distinguish them from the verified and uncharacterized (henceforth called validated) ORFs. However, our analysis shows that 84.2% of the validated ORFs and only 25% of the dubious ORFs have more stable sense than antisense RNA/DNA duplexes (Figure 2B). This ratio depends on ORF length and is 90.35% for ORFs longer than 2000 bp and only 45.29% for ORFs shorter than 250 bp (Table 4). These data suggest a way to distinguish true from spurious ORFs based solely on their thermodynamic stability profiles. To test this proposition, we extended our analysis to all potential ORFs found in the other three *Saccharomyces species* (*S. bayanus, S. paradoxus* and *S. mikatae*). We took advantage of the fact that ORFs in these genomes that have orthologs in *S. cerevisiae* were identified by comparative genomic analysis, assuming these ORFs to be true [14], [15]. We found that more than 81% of the true ORFs and only 28.5% of the spurious genes have more stable sense than antisense RNA/DNA duplexes. Therefore, false positives and negatives under our thermodynamic approach are 19% and 28.5%, respectively. In addition, the length dependence of sense/antisense duplex stability in these three species is reminiscent of the one observed in *S. cerevisiae*-more than 90% of the true ORFs longer than 2000 bp and less than 61% of the true ORFs shorter than 250 bp have more stable sense than antisense RNA/DNA duplex. These results further strengthen the idea that thermodynamic stability is able to discriminate to a certain extent between true and spurious ORFs.

The genome of *S. cerevisiae* contains 1204 annotated overlapping ORFs grouped in 634 overlapping pairs (Table S6). 91% of the groups consist of both verified and dubious ORFs and less than 5% of these groups contain only validated ORFs suggesting that *S. cerevisiae* does not tolerate overlapping mRNA transcription. To examine whether the stability of mRNA/DNA duplexes influences the choice of ORF to be transcribed, we compared the stability profiles of the groups containing both dubious and validated ORFs. In 81.5% of the cases, validated ORFs have more stable sense mRNA/DNA duplex than the dubious ORFs, determining to an extent which of the ORFs is to be transcribed.

Furthermore, we looked into the thermodynamic profiles of genes containing introns. Our results show that in contrast to exons, introns have less stable sense RNA/DNA duplex than the respective antisense RNA/DNA duplex (Table S3). Therefore, more stable sense than potential antisense RNA/DNA duplexes are characteristic of the coding sequences.

### Differential distribution of certain nucleotide neighbor interactions in sense and antisense RNA/DNA duplexes is responsible for the higher thermodynamic stability of sense RNA/DNA duplexes of coding sequences

**Figure 4:**
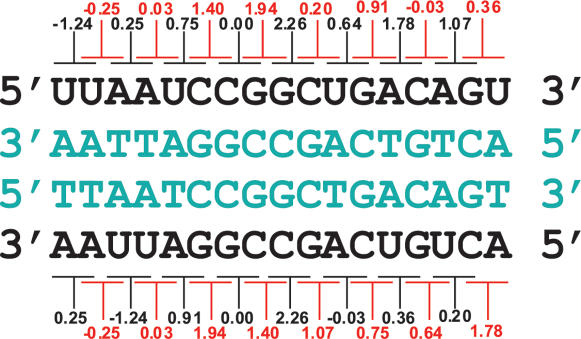
Thermodynamic stability (ΔG) of the nearest-neighbor interactions in RNA/DNA duplexes (10 mM monovalent cation), containing complementary DNA strands (in blue). Watson strand (top) and Crick strand (bottom) shown in black.

To explain the observed differences in the stability of sense and potential antisense RNA/DNA duplexes in coding sequences and introns, we calculated the frequency of their nearest neighbor interactions. RNA/DNA nearest neighbor interactions form pairs, containing complementary DNA duplets (Figure 4). Differences in ΔG values of for interactions within these pairs are responsible for the difference in stability of sense/antisense duplexes. We found that genes' sense mRNA/DNA duplexes contain more rAA/dTT, rAC/dTG, rAG/dTC, rGG/dCC, rGA/dCT, rCA/dGT interactions than their corresponding partners rUU/dAA, rGU/dCA, rCU/dGA, rCC/dGG, rUC/dAG, rUG/dAC found more frequently in the potential antisense RNA/DNA duplexes (Table S7). The higher stability of the first five sense interactions (rAA/dTT, rAC/dTG, rAG/dTC, rGG/dCC, rUC/dAG) compared to the corresponding antisense partners (rUU/dAA, rGU/dCA, rCU/dGA, rCC/dGG, rGA/dCT) leads to a more stable sense RNA/DNA duplex. rUG/dAC is more stable and well-represented in antisense duplexes than rCA/dGT and hence it contributes to the stability of the antisense duplex. Finally, rAU/dTA and rUA/dAT, as well as rGC/dCG and rCG/dGC, are symmetric and therefore equally distributed in both sense and antisense duplexes and contribute equally to their stability. Yet, the impact of the first five duplex pairs on the stability of the sense duplex is much stronger and consequently sense duplexes are more stable than antisense duplexes. In introns and IGRs, however, the above frequencies are different (Tables S8 and S9). For example, in contrast to coding sequences, the more stable rAA/dTT pair is under-represented in introns compared to its corresponding but less stable rUU/dAA pair. These two pairs occur with nearly equal frequency in IGRs. This suggests that the different distribution of certain nearest neighbor interactions contributes to the higher stability of coding sequences and lower stability of introns and IGRs.

## Discussion

It still remains unclear how mRNA/DNA duplexes stability influences mRNA level. The co-transcriptional nature of 3′-end processing provides an elegant possible explanation [16]. The 3′-end processing machinery, traveling along RNA polymerase II recognizes the 3′-end processing sites within the nascent mRNA and catalyzes endonucleolytic cleavage and addition of poly(A) tail. An important factor here is the rate and extent of mRNA/DNA duplex unwinding immediately after mRNA synthesis. Slower and inefficient unwinding of mRNA/DNA duplex in the 3′-end processing region will hinder its recognition by the 3′-end processing machinery. Therefore, in regions of higher stability where RNA/DNA duplexes are more difficult to unwind and less accessible to the processing apparatus RNA processing will be impaired. A similar mechanism could act during splicing. Introns are known to harbor common (even though very degenerate) RNA consensus sequences near their 3′ and 5′-ends that are recognized and cleaved by spliceosomal components to remove introns and ligate flanking exon sequences. Again, a critical step is the recognition of these elements by the spliceosome traveling with the RNA polymerase II [16], [17]. Hence, the lower thermodynamic stability of mRNA/DNA duplex within introns will make consensus splicing sequences more accessible and easier to recognize, thus improving splicing efficiency. If this model is correct, the higher thermodynamic stability of mRNA/DNA duplexes in the genes' coding sequences would preserve mRNA from premature termination and improper splicing.

The above model is challenged in the light of the fact that the length of mRNA/DNA duplex during transcription is considered to be only 7–9 bp and is located within the polymerase enzyme [18]. However, these estimates are derived from biochemical assays of stalled transcription complexes [18]. Static transcriptional machinery gives enough time for re-association of the DNA/DNA helix outside the polymerase. Such re-association can restrict the length of the mRNA/DNA duplex to be maintained by the RNA pol II. Supporting this idea are experiments showing that mRNA/DNA duplex is not unwound by RNA polymerase when the non-template DNA strand is missing [19]. Addition of non-template DNA strand restricts the mRNA/DNA duplex to 9 nucleotides [19]. However, the length of the mRNA/DNA duplex would be different in case of dynamic RNA polymerase and would strongly depend on RNA/DNA, DNA/DNA stability and the rate of RNA polymerase movement. More stable mRNA/DNA duplexes would persist longer outside the polymerase. In addition, during transcription, negative superstress is generated behind the Pol II enzyme [20] which should temporarily impede the re-association of the two DNA strands and would thus slow down mRNA/DNA duplex unwinding. The influence of RNA/DNA stability on RNA/DNA duplex length could give a reasonable explanation of the differences between the two atomic structures of the RNA polymerase complex containing RNA/DNA duplex. In one of the studies, the RNA/DNA duplex is unwound at the RNA's 5′-end [21] while in the other it is not [22]. In the first experiment, the last three nucleotides at the 5′-RNA end are AUG, forming two of the less stable nearest neighbor interactions rAU/dTA (0.03 kcal/mol) and rUG/dAC (0.64 kcal/mol) which allow RNA unwinding by two protein loops (named lid and rudder) of Pol II. In the second experiment, the 5′-end of the RNA strand contains three G residues that participate in two rGG/dCC nearest neighbor interactions. These residues form the second most thermodynamically stable RNA/DNA duplex structure (1.94 kcal/mol) which would prevent the lid and the rudder from unwinding RNA.

In addition, DNA/DNA and mRNA/DNA duplex stability could affect mRNA level by influencing the kinetics of transcription. It has been suggested that the free energy required to open the DNA transcription bubble and to form the mRNA/DNA hybrid directly influences the rate of transcription elongation [23], [24]. It has been shown that transcription machinery tends to pause when the mRNA/DNA hybrid is unstable [25]. Pausing or rate reduction at unstable mRNA/DNA duplexes of 3′-UTRs and introns could give enough time to the processing complexes to interact with their corresponding mRNA elements and process the nascent mRNA transcript. Likewise, the higher stability of mRNA/DNA duplexes of the coding sequences could increase the rate of the transcription elongation and raise mRNA level.

In this work we have shown that DNA/DNA as well as RNA/DNA duplex stability differ between coding and non-coding regions. Moreover, sense RNA/DNA duplexes appear to be more stable than the corresponding anti-sense duplexes, an observation potentially useful for gene discovery. The lower stability of the DNA/DNA and mRNA/DNA duplexes of 3′-untranslated regions and higher stability of the coding sequences correlate with increased mRNA level. Our results suggest that the thermodynamic stability of DNA/DNA and mRNA/DNA duplexes affects mRNA transcription but further work will be required to more fully understand how thermodynamic stability modulates mRNA level.

## Materials and Methods

### Genomes and annotations

The complete genome sequence of *S. cerevisiae* (SGD release 07.2005) strain S288C [26] and the draft genomes of *S. bayanus, S. mikatae* and *S. paradoxus* [13] were used in the calculations. 3′-IGR, which do not overlap with coding sequences, of all four *Saccharomyces* species, were analyzed. In *S. bayanus, S. mikatae* and *S. paradoxus* we used the full-length ORFs only. For these three *Saccharomyces* species only the 3′-IGRs surrounded by full-length ORFs, with orthologs in *S. cerevisiae'*s, and belonging to a common contig were included in the analysis.

### Measurement of thermodynamic stability

ΔG of the nearest-neighbor interactions was calculated by Perl-based software (supplementary Data S1) using Kowalski's sliding-window approach [6]. Published values of ΔH and ΔS for each nearest-neighbor interaction for DNA/DNA duplex [2] and RNA/DNA duplex [3] were used. Our analysis does not consider the possible self-folding of the single stranded DNA and RNA as in living systems the processes of DNA unwinding and RNA synthesis are independent of RNA and DNA self-folding. During transcription, DNA unwinding is clearly separated from the self-folding of the single stranded DNA and is carried out by the helicase activity of RNA polymerase II holoenzyme in 5′-3′ orientation one nucleotide at a time [24]. Therefore, to allow self-folding of a palindromic sequence of six nucleotides, six independent DNA unwinding reactions are required. After that, RNA polymerase II adds ribonucleotides one by one and creates an RNA/DNA duplex. Therefore, measurements of RNA/DNA duplex stability do not require the consideration of RNA or DNA self-folding as RNA is synthesized not by annealing of oligonucleotides (that could self-fold) but by sequential addition of ribonucleotides to the nascent transcript.

Calculations are carried out for 37°C, with a step size of 1 bp and a window size of 100 bp, 9 bp or 2 bp. The calculated values for different window sizes are indicated at the 51^st^ bp for 100 bp windows, at the 5^th^ bp for 9 bp windows, and at the 2^nd^ bp for 2 bp windows. A 2-bp window represents a single nearest-neighbor interaction. Window size of 9 bp allows calculation of ΔG for sequences equal in size to the length of the RNA/DNA duplex maintained by RNA polymerase II during transcription elongation [18]. Window size of 100 bp enables calculation of ΔG average of the windows that extend over large genomic regions. Our results show that there is no significant difference in the ratio of ΔG avg of genes and intergenic regions when calculations were carried out using different window sizes (Table S1 and S4). In addition, there is no significant difference in both the ratio of ΔG average of sense/antisense RNA/DNA duplexes and the correlation between ΔG and mRNA level, using different window sizes. Therefore, we generally used a window size of 100 bp, except for introns and UTRs (window size of 9 bp used instead) as they tend to be relatively short.

ΔG was calculated for three different salt concentrations (10mM, 100mM and 1M) [6], [27], [28]. No significant differences in both the ratio of ΔG avg of genes and intergenic regions and ΔG avg of sense/antisense RNA/DNA duplexes were observed (Table S1 and S4). The results presented in this work assume monovalent cation concentration of 10mM as this is the value used in previous studies on thermodynamic stability of DNA/DNA duplexes [6], [29].

Stability of RNA/DNA duplexes of both DNA strands was calculated over the entire genomes. Thermodynamic stability of sense RNA/DNA duplexes for genes was calculated using duplexes containing gene's template DNA strand and stability of antisense RNA/DNA duplexes was calculated using duplexes containing gene's coding DNA strand.

### Statistics

Spearman's rank correlation test was used to assess the relationship between either DNA/DNA or mRNA/DNA duplex stability and mRNA level. Variation of Spearman's correlation coefficient from 0 to 1 indicates that the two variables increase together and from 0 to-1 indicates negative relationship. Wilcoxon–Mann–Whitney rank sum test was used to statistically evaluate the difference between genes' and IGRs' ΔG avg and ΔG min in DNA/DNA and mRNA/DNA duplexes and evaluate the difference between genes' ΔG avg in sense and antisense RNA/DNA duplexes.

### Supporting web site

Supporting web site (http://obzor.bio21.bas.bg/stoyno/) contains: (i) all raw thermodynamic stability data, (ii) the software and databases used for ΔG calculation and (iii) plots, presenting DNA/DNA and RNA/DNA duplex stability of all sixteen chromosomes of *S. cerevisiae*.

## Supporting Information

Data S1 Method of thermodynamic stability measurement
(0.03 MB DOC)

Table S1 Delta G values in DNA/DNA and RNA/DNA duplexes of genes, introns, exons, UTRs and EPRs
(4.66 MB ZIP)

Table S2 Free energy minimums in DNA/DNA duplexes of intergenic regions flanked by convergent, divergent and tandem running transcripts.
(0.31 MB ZIP)

Table S3 Estimation of statistically significant difference
(0.04 MB DOC)

Table S4 Comparison between values of delta G average and delta G minimum of the genes and intergenic regions adjacent to their 3′ ends
(3.19 MB ZIP)

Table S5 Correlation between mRNA level and thermodynamic stability of DNA/DNA and RNA/DNA duplexes
(0.08 MB DOC)

Table S6 Comparison between delta G average of the overlapping ORF couples in sense and antisense RNA/DNA dupexes
(0.07 MB ZIP)

Table S7 Distribution of the nearest-neighbor interactions in sense and antisense RNA/DNA duplexes in genes
(0.04 MB DOC)

Table S8 Distribution of the nearest-neighbor interactions in sense and antisense RNA/DNA duplexes in 3′-IGRs
(0.04 MB DOC)

Table S9 Distribution of the nearest-neighbor interactions in sense and antisense RNA/DNA duplexes in introns
(0.04 MB DOC)
