# Supplementary material for: Stability of mRNA/DNA and DNA/DNA Duplexes Affects mRNA Transcription
Source: PLoS One. 2007 Mar 14;2(3):e290. doi: 10.1371/journal.pone.0000290 (PMC1808433; doi:10.1371/journal.pone.0000290)
Supplement: Data S1 — Method of thermodynamic stability measurement (0.03 MB DOC) [file pone.0000290.s001.doc]

**sUPPLEMENTARY DATA**

**Measurement of thermodynamic stability**

In our analysis we used a combination of sliding-window approach and a nearest-neighbor method.

ΔG of the nearest-neighbor interactions was calculated by Perl-based software using the following equation:

ΔG° = ΔH° [1 - (T/Tm)], where:

ΔH° = sum of ΔH° for each of the nearest neighbor interactions in a given window

ΔS° = sum of ΔS° for each of the nearest neighbor interactions in a given window

Tm = (ΔH°/ΔS°) + 18 log [monovalent cation]
