# Supplementary material for: Stability of mRNA/DNA and DNA/DNA Duplexes Affects mRNA Transcription
Source: PLoS One. 2007 Mar 14;2(3):e290. doi: 10.1371/journal.pone.0000290 (PMC1808433; doi:10.1371/journal.pone.0000290)
Supplement: Table S3 — Estimation of statistically significant difference (0.04 MB DOC) [file pone.0000290.s004.doc]

**Table S3.** Estimation of statistically significant difference between thermodynamic stability of different features and between thermodynamic stability of sense and antisense RNA/DNA duplexes, measured by Mann-Whitney Rank Sum Test. The difference between two groups below P = 0.05 is greater than expected by chance.

|  | Statistically significant difference (P- value) | Total number of features |
| --- | --- | --- |
| DNA/DNA ΔG avg Genes-IGR | P < 0.0000000001 | 6562 - 5974 |
| DNA/DNA ΔG avg IGR- divergent IGR | P < 0.0000000001 | 5947 - 1491 |
| DNA/DNA ΔG avg IGR- convergent IGR | P < 0.0000000001 | 5947 - 1571 |
| DNA/DNA ΔG avg IGR- tandem IGR | P < 0.0000000001 | 5947 - 2854 |
| DNA/DNA ΔG avg Genes-3’IGR | P < 0.0000000001 | 6004 - 6004 |
| mRNA/DNA ΔG avg Genes-3’IGR | P < 0.0000000001 | 6004 - 6004 |
| DNA/DNA Genes ΔG avg -3’EPR ΔG | P < 0.0000000001 | 2995 - 2995 |
| mRNA/DNA Genes ΔG avg -3’EPR ΔG | P < 0.0000000001 | 2995 - 2995 |
| DNA/DNA ΔG avg Genes -3’UTR | P < 0.0000000001 | 2995 - 2995 |
| mRNA/DNA ΔG avg Genes -3’UTR | P < 0.0000000001 | 2995 - 2995 |
| DNA/DNA ΔG avg Genes -Introns | P < 0.0000000001 | 264 - 264 |
| mRNA/DNA ΔG avg Genes -Introns | P < 0.0000000001 | 264 - 264 |
| DNA/DNA ΔG avg Exons -Introns | P < 0.0000000001 | 264 - 264 |
| mRNA/DNA ΔG avg Exons -Introns | P < 0.0000000001 | 264 - 264 |
| Genes ΔG avg sense-antisense RNA/DNA | P < 0.0000000001 | 6562 - 6562 |
| 3’IGR ΔG avg sense-antisense RNA/DNA | P = 0.044 | 6004 - 6004 |
| 3’EPR ΔG avg sense-antisense RNA/DNA | P < 0.0000000001 | 3010 - 3010 |
| 3’UTR ΔG avg sense-antisense RNA/DNA | P < 0.0000000001 | 2995 - 2995 |
| Introns ΔG avg sense-antisense RNA/DNA | P < 0.0000000001 | 264 - 264 |
| Exons ΔG avg sense-antisense RNA/DNA | P < 0.0000000001 | 264 - 264 |
