# Supplementary material for: Stability of mRNA/DNA and DNA/DNA Duplexes Affects mRNA Transcription
Source: PLoS One. 2007 Mar 14;2(3):e290. doi: 10.1371/journal.pone.0000290 (PMC1808433; doi:10.1371/journal.pone.0000290)
Supplement: Table S5 — Correlation between mRNA level and thermodynamic stability of DNA/DNA and RNA/DNA duplexes (0.08 MB DOC) [file pone.0000290.s006.doc]

**Table S5.** Correlation between mRNA level and thermodynamic stability of different features, measured by Spearman’s rank correlation test and their dependence on ORF length. For pairs with P values greater than 0.05, there is no significant relationship between the two variables (indicated by *).

|  | | Length (bp) | Spearman’s rank correlation coefficient | Statistical significance  (P-value) | Total number of features |
| --- | --- | --- | --- | --- | --- |
| Genes ΔG avg window 100 | DNA/DNA | All genes | 0.209 | P < 0.0000000001 | 4969 |
|  |  | Genes > 2 000 | 0.266 | P < 0.0000000001 | 1150 |
|  | mRNA/DNA sense | All genes | 0.142 | P < 0.0000000001 | 4969 |
|  |  | Genes > 2 000 | 0.136 | P < 0.0000000001 | 1150 |
|  | RNA/DNA antisense | All genes | 0.241 | P < 0.0000000001 | 4969 |
|  |  | Genes > 2 000 | 0.302 | P < 0.0000000001 | 1150 |
| Genes ΔG avg window 9 | DNA/DNA | All genes | 0.222 | P < 0.0000000001 | 4969 |
|  | mRNA/DNA sense | All genes | 0.151 | P < 0.0000000001 | 4969 |
|  | RNA/DNA antisense | All genes | 0.254 | P < 0.0000000001 | 4969 |
| ΔG avg of coding sequences window 9 | DNA/DNA | All genes | 0.263 | P < 0.0000000001 | 4969 |
|  | mRNA/DNA sense | All genes | 0.199 | P < 0.0000000001 | 4969 |
|  | RNA/DNA antisense | All genes | 0.283 | P < 0.0000000001 | 4969 |
| ΔG avg of intron containing genes window 9 | DNA/DNA | All genes |  | P = 0.887 * | 198 |
|  | mRNA/DNA sense | All genes |  | P = 0.422 * | 198 |
|  | RNA/DNA antisense | All genes |  | P = 0.378 * | 198 |
| ΔG avg of coding sequences in intron containing ORFs window 9 | DNA/DNA | All genes | 0.374 | P < 0.0000000001 | 198 |
|  |  | Genes > 2 000 | 0.658 | P < 0.0000000001 | 24 |
|  | mRNA/DNA sense | All genes | 0.329 | P < 0.0000000001 | 198 |
|  |  | Genes > 2 000 | 0.691 | P < 0.0000000001 | 24 |
|  | RNA/DNA antisense | All genes | 0.493 | P < 0.0000000001 | 198 |
|  |  | Genes > 2 000 | 0.758 | P < 0.0000000001 | 24 |
| Introns ΔG avg window 9 | DNA/DNA | All genes | 0.275 | P < 0.0000000001 | 198 |
|  |  | Genes > 2 000 | 0.611 | P = 0.00155 | 24 |
|  | mRNA/DNA sense | All genes | 0.319 | P < 0.0000000001 | 198 |
|  |  | Genes > 2 000 | 0.560 | P = 0.00449 | 24 |
|  | RNA/DNA antisense | All genes | -0.199 | P = 0.00497 | 198 |
|  |  | Genes > 2 000 | 0.609 | P = 0.00164 | 24 |
| 3’-EPR ΔG window 100 | DNA/DNA | All genes | - 0.266 | P < 0.0000000001 | 2756 |
|  |  | Genes < 250 | -0.427 | P = 0.0329 | 25 |
|  |  | Genes < 500 | -0.413 | P < 0.0000000001 | 270 |
|  |  | Genes > 2 000 | -0.156 | P < 0.0000000001 | 587 |
|  | mRNA/DNA sense | All genes | - 0.231 | P < 0.0000000001 | 2756 |
|  |  | Genes < 250 | -0.639 | P < 0.0000000001 | 25 |
|  |  | Genes < 500 | -0.400 | P < 0.0000000001 | 270 |
|  |  | Genes > 2 000 | -0.110 | P = 0.00768 | 587 |
|  | RNA/DNA antisense | All genes | -0.134 | P < 0.0000000001 | 2756 |
|  |  | Genes < 250 |  | P = 0.609* | 25 |
|  |  | Genes < 500 | -0.279 | P < 0.0000000001 | 270 |
|  |  | Genes > 2 000 | -0.108 | P = 0.00861 | 587 |
| 3-UTR ΔG avg window 9 | DNA/DNA | All genes | -0.220 | P < 0.0000000001 | 2745 |
|  |  | Genes < 250 | -0.417 | P = 0.0380 | 25 |
|  |  | Genes < 500 | -0.359 | P < 0.0000000001 | 270 |
|  |  | Genes > 2 000 | -0.173 | P < 0.0000000001 | 584 |
|  | mRNA/DNA sense | All genes | - 0.195 | P < 0.0000000001 | 2745 |
|  |  | Genes < 250 | - 0.648 | P < 0.0000000001 | 25 |
|  |  | Genes < 500 | -0.434 | P < 0.0000000001 | 270 |
|  |  | Genes > 2 000 | -0.134 | P = 0.00118 | 584 |
|  | RNA/DNA antisense | All genes | - 0.144 | P < 0.0000000001 | 2745 |
|  |  | Genes < 250 |  | P = 0.263 * | 25 |
|  |  | Genes < 500 | -0.257 | P < 0.0000000001 | 270 |
|  |  | Genes > 2 000 | -0.129 | P = 0.00185 | 584 |
