# Supplementary material for: Stability of mRNA/DNA and DNA/DNA Duplexes Affects mRNA Transcription
Source: PLoS One. 2007 Mar 14;2(3):e290. doi: 10.1371/journal.pone.0000290 (PMC1808433; doi:10.1371/journal.pone.0000290)
Supplement: Table S7 — Distribution of the nearest-neighbor interactions in sense and antisense RNA/DNA duplexes in genes (0.04 MB DOC) [file pone.0000290.s008.doc]

**Table S7.** Distribution of the nearest-neighbor interactions (NN) in sense and antisense RNA/DNA duplexes in genes. Positive contribution of the NN pairs to more stable sense than antisense duplex was indicated (+),negative contribution was indicated (-) respectively.

| **NN** | **Number of NN in sense duplexes** | **Number of NN in antisense duplexes** | **% of NN in sense duplexes** | **% of NN in antisense duplexes** | **DeltaG of NN (0.01M NaCl)** | **Difference in number of NN in sense and antisense duplexes** | **Contribution of the NN pairs to more stable sense than antisense duplex** |
| --- | --- | --- | --- | --- | --- | --- | --- |
| **rAA/dTT** | 1026948 | 794568 | 11.66908 | 9.028575 | 0.25 | 232380 | + |
| **rUU/dAA** | 794568 | 1026948 | 9.028575 | 11.66908 | -1.24 | -232380 |
| **rAU/dTA** | 754415 | 754415 | 8.572322 | 8.572322 | 0.03 | 0 |  |
| **rUA/dAT** | 579668 | 579668 | 6.586694 | 6.586694 | -0.25 | 0 |
| **rCA/dGT** | 601809 | 584049 | 6.838279 | 6.636475 | -0.03 | 17760 | - |
| **rUG/dAC** | 584049 | 601809 | 6.636475 | 6.838279 | 0.64 | -17760 |
| **rGU/dCA** | 428650 | 503508 | 4.870696 | 5.721298 | 0.36 | -74858 | + |
| **rAC/dTG** | 503508 | 428650 | 5.721298 | 4.870696 | 1.78 | 74858 |
| **rCU/dGA** | 473350 | 573827 | 5.378616 | 6.520323 | 0.2 | -100477 | + |
| **rAG/dTC** | 573827 | 473350 | 6.520323 | 5.378616 | 1.07 | 100477 |
| **rGA/dCT** | 650023 | 492560 | 7.386129 | 5.596897 | 0.91 | 157463 | + |
| **rUC/dAG** | 492560 | 650023 | 5.596897 | 7.386129 | 0.75 | -157463 |
| **rCG/dGC** | 263050 | 263050 | 2.989004 | 2.989004 | 0 | 0 |  |
| **rGC/dCG** | 342025 | 342025 | 3.886387 | 3.886387 | 2.26 | 0 |
| **rGG/dCC** | 386058 | 346083 | 4.386728 | 3.932497 | 1.94 | 39975 | + |
| **rCC/dGG** | 346083 | 386058 | 3.932497 | 4.386728 | 1.4 | -39975 |
