# Supplementary material for: Stability of mRNA/DNA and DNA/DNA Duplexes Affects mRNA Transcription
Source: PLoS One. 2007 Mar 14;2(3):e290. doi: 10.1371/journal.pone.0000290 (PMC1808433; doi:10.1371/journal.pone.0000290)
Supplement: Table S8 — Distribution of the nearest-neighbor interactions in sense and antisense RNA/DNA duplexes in 3′-IGRs (0.04 MB DOC) [file pone.0000290.s009.doc]

**Table S8.** Distribution of the nearest-neighbor interactions in sense and antisense RNA/DNA duplexes in 3’-IGRs. Positive contribution of the NN pairs to more stable sense than antisense duplex was indicated (+),negative contribution was indicated (-) respectively.

| **NN** | **Number of NN in sense duplexes** | **Number of NN in antisense duplexes** | **% of NN in sense duplexes** | **% of NN in antisense duplexes** | **DeltaG of NN (0.01M NaCl)** | **Difference in number of NN in sense and antisense duplexes** | **Contribution of the NN pairs to more stable sense than antisense duplex** |
| --- | --- | --- | --- | --- | --- | --- | --- |
| **rAA/dTT** | 377458 | 382711 | 11.9543 | 12.1206 | 0.25 | -5253 | - |
| **rUU/dAA** | 382711 | 377458 | 12.1206 | 11.9543 | -1.24 | 5253 |
| **rAU/dTA** | 325238 | 325238 | 10.3004 | 10.3004 | 0.03 | 0 |  |
| **rUA/dAT** | 301179 | 301179 | 9.53852 | 9.53852 | -0.25 | 0 |
| **rCA/dGT** | 188269 | 179187 | 5.96259 | 5.67496 | -0.03 | 9082 | - |
| **rUG/dAC** | 179187 | 188269 | 5.67496 | 5.96259 | 0.64 | -9082 |
| **rGU/dCA** | 161097 | 166020 | 5.10204 | 5.25795 | 0.36 | -4923 | + |
| **rAC/dTG** | 166020 | 161097 | 5.25795 | 5.10204 | 1.78 | 4923 |
| **rCU/dGA** | 172912 | 171415 | 5.47622 | 5.42881 | 0.2 | 1497 | - |
| **rAG/dTC** | 171415 | 172912 | 5.42881 | 5.47622 | 1.07 | -1497 |
| **rGA/dCT** | 171598 | 177109 | 5.43461 | 5.60915 | 0.91 | -5511 | - |
| **rUC/dAG** | 177109 | 171598 | 5.60915 | 5.43461 | 0.75 | 5511 |
| **rCG/dGC** | 84576 | 84576 | 2.67857 | 2.67857 | 0 | 0 |  |
| **rGC/dCG** | 103232 | 103232 | 3.26942 | 3.26942 | 2.26 | 0 |
| **rGG/dCC** | 96139 | 99361 | 3.04478 | 3.14682 | 1.94 | -3222 | - |
| **rCC/dGG** | 99361 | 96139 | 3.14682 | 3.04478 | 1.4 | 3222 |
