# Supplementary material for: Stability of mRNA/DNA and DNA/DNA Duplexes Affects mRNA Transcription
Source: PLoS One. 2007 Mar 14;2(3):e290. doi: 10.1371/journal.pone.0000290 (PMC1808433; doi:10.1371/journal.pone.0000290)
Supplement: Table S9 — Distribution of the nearest-neighbor interactions in sense and antisense RNA/DNA duplexes in introns (0.04 MB DOC) [file pone.0000290.s010.doc]

**Table S9.** Distribution of the nearest-neighbor interactions in sense and antisense RNA/DNA duplexes in introns. Positive contribution of the NN pairs to more stable sense than antisense duplex was indicated (+),negative contribution was indicated (-) respectively.

| **NN** | **Number of NN in sense duplexes** | **Number of NN in antisense duplexes** | **% of NN in sense duplexes** | **% of NN in antisense duplexes** | **DeltaG of NN (0.01M NaCl)** | **Difference in number of NN in sense and antisense duplexes** | **Contribution of the NN pairs to more stable sense than antisense duplex** |
| --- | --- | --- | --- | --- | --- | --- | --- |
| **rAA/dTT** | 7493 | 8631 | 11.46894 | 13.21078 | 0.25 | -1138 | - |
| **rUU/dAA** | 8631 | 7493 | 13.21078 | 11.46894 | -1.24 | 1138 |
| **rAU/dTA** | 6473 | 6473 | 9.907704 | 9.907704 | 0.03 | 0 |  |
| **rUA/dAT** | 6000 | 6000 | 9.18372 | 9.18372 | -0.25 | 0 |
| **rCA/dGT** | 3759 | 4111 | 5.753601 | 6.292379 | -0.03 | -352 | + |
| **rUG/dAC** | 4111 | 3759 | 6.292379 | 5.753601 | 0.64 | 352 |
| **rGU/dCA** | 3446 | 3618 | 5.274517 | 5.537783 | 0.36 | -172 | + |
| **rAC/dTG** | 3618 | 3446 | 5.537783 | 5.274517 | 1.78 | 172 |
| **rCU/dGA** | 3536 | 3624 | 5.412273 | 5.546967 | 0.2 | -88 | + |
| **rAG/dTC** | 3624 | 3536 | 5.546967 | 5.412273 | 1.07 | 88 |
| **rGA/dCT** | 3965 | 3333 | 6.068909 | 5.101557 | 0.91 | 632 | + |
| **rUC/dAG** | 3333 | 3965 | 5.101557 | 6.068909 | 0.75 | -632 |
| **rCG/dGC** | 1602 | 1602 | 2.452053 | 2.452053 | 0 | 0 |  |
| **rGC/dCG** | 1946 | 1946 | 2.978587 | 2.978587 | 2.26 | 0 |
| **rGG/dCC** | 1927 | 1869 | 2.949505 | 2.860729 | 1.94 | 58 | + |
| **rCC/dGG** | 1869 | 1927 | 2.860729 | 2.949505 | 1.4 | -58 |
